# Improving Procedural Confidence: Outcomes of a Surgical Skills Workshop for Medical Students

**DOI:** 10.7759/cureus.111386

**Published:** 2026-06-23

**Authors:** Mary Arden G Guillory, Jennifer Escamilla, Anika Doppalapudi, Ashlyn G Holubar, Kaitlyn D Ybanez, Mark Manickath, Maci Oestreich, Mckenna Stallworth, Kelsey Baker

**Affiliations:** 1 Medicine, University of Texas Rio Grande Valley, Edinburg, USA; 2 Medicine, University of Texas Medical Branch at Galveston, Galveston, USA; 3 Neuroscience, University of Texas Rio Grande Valley, Edinburg, USA

**Keywords:** early clinical exposure, medical simulation training, preclinical medical education, procedural skills confidence, simulation-based education, surgical career interest, undergraduate medical education, workshop-based learning

## Abstract

Introduction

Introducing surgical skills early in medical training may enhance students’ confidence and readiness for clinical training. Simulation-based skill workshops provide students with a comfortable learning environment, resulting in effective learning. The goal of the Surgical Skills Workshop was to provide first- and second-year medical students with hands-on exposure to essential surgical skills. The educational impact on medical students' confidence in performing the respective surgical skills and changes in surgical career interest was assessed.

Methods

First- and second-year medical students at the University of Texas Rio Grande Valley School of Medicine participated in a hands-on surgical skills workshop comprising eight stations: endotracheal intubation, suturing, sterile scrubbing and gowning, Foley catheter placement, venipuncture, intrauterine device insertion, laparoscopic simulation, and nasogastric tube insertion. Participants rotated through stations in small groups with instruction from residents and attending physicians. Pre- and post-workshop surveys assessed confidence in the respective surgical skill using a five-point Likert scale, as well as interest in a surgical career. Statistical comparisons were performed, with P <0.01 considered significant.

Results

Fifty-one students completed the pre-workshop survey, and 46 completed the post-workshop survey, with a response rate of 90%. Self-reported confidence increased significantly across nearly all assessed procedural skills following the workshop (P<0.01). The largest gains were observed in skills typically unfamiliar to preclinical students, including sterile scrubbing and gowning, nasogastric and endotracheal tube placement, and laparoscopic instrument handling. Interest in pursuing a surgical career did not change significantly following the event, likely because participants demonstrated high baseline interest prior to participation. However, the survey aimed not only to assess changes in interest level, but also whether early hands-on exposure to surgical skills reinforced, clarified, or negatively impacted students’ perceptions of a surgical career. The absence of a decline in interest following realistic procedural exposure may suggest that the workshop helped consolidate pre-existing interest rather than substantially alter career intentions.

Conclusions

The Surgical Skills Workshop significantly improved preclinical medical students' perceived confidence across a broad range of surgical skills. While exposure to various surgical skills did not affect career interest, the simulation-based nature of the workshop fostered readiness for clinical clerkships. Future studies should incorporate objective performance measures to assess skill competence and broader student participation to better assess long-term career impacts.

## Introduction

Early exposure to surgical skills during medical training has been shown to positively influence medical students’ confidence, preparedness, and interest in pursuing surgical careers [1]. A systematic review examining preclerkship surgical skills workshops reported that two-thirds of participating students (8 of 12) demonstrated increased interest in surgical specialties, which was attributed to improved preparedness and more favorable perceptions of surgery as a career option [1]. These findings highlight the potential role of early, structured procedural exposure in shaping career decision-making among medical students.

Beyond career interest, surgical skills workshops have consistently demonstrated educational benefits [2,3]. Prior studies have shown significant improvements in both self-reported and faculty-assessed confidence among medical students following participation in surgical knot-tying and basic procedural skills training [4-6]. Additionally, intensive surgical bootcamps have been associated with improved procedural competency at the clerkship level, as measured by objective assessments such as videotaped Objective Structured Assessment of Technical Skills (OSATS) [7]. Although our study was based on a subjective student survey, the aforementioned study showed that surgical bootcamps improved competency. Students completing these bootcamps have also demonstrated measurable gains in instrument handling, dexterity, and technical precision, suggesting that simulation-based education can effectively enhance foundational surgical skills before formal clinical exposure [8]. Exposure to laparoscopic simulation training allows medical students to gain earlier experience with contemporary surgical approaches [2,3,9].

Simulation-based procedural training provides a low-risk, supportive learning environment that allows students to practice technical skills, receive immediate feedback, and develop comfort with clinical procedures prior to entering patient-care settings. Faculty-supported, hands-on workshops further enhance this learning experience by combining expert instruction with peer collaboration and deliberate practice. Despite growing evidence supporting the value of these interventions, variability remains in workshop structure, skill selection, and educational outcomes, underscoring the need for continued evaluation of innovative surgical skills programs.

To address this need, the Surgical Skills Workshop was developed and implemented at the University of Texas Rio Grande Valley School of Medicine (UTRGVSOM) as an interactive, faculty-supported, and student-led procedural training event. The workshop was designed to expose students to a broad range of clinically relevant procedural skills through eight hands-on stations: (1) intubation, (2) suturing, (3) sterile scrubbing and gowning, (4) Foley catheter placement, (5) venipuncture, (6) intrauterine device (IUD) insertion, (7) laparoscopic simulation, and (8) nasogastric tube insertion. Each station was led by a third-year medical student, resident physician, or attending physician with relevant clinical expertise. Students were randomly assigned to small groups and rotated through stations at 25-minute intervals, allowing for active participation, collaboration, and real-time individualized feedback. This study aims to evaluate the educational impact of the Surgical Skills Workshop on medical students’ confidence in performing procedural skills and their interest in pursuing surgical careers. By assessing student-reported outcomes following participation in a structured, simulation-based workshop, this study seeks to further elucidate the role of early procedural exposure in undergraduate medical education.

## Materials and methods

Study design

A cohort survey of medical students attending the University of Texas Rio Grande Valley School of Medicine was conducted to assess the effectiveness of a surgical skills workshop in promoting an interest among medical students in a future surgical career. The surgical skills event took place at the University of Texas Rio Grande Valley School of Medicine Clinical Skills Center and was led by general surgery residents and attending physicians, a urology attending physician, an obstetrics and gynecology (OBGYN) attending physician, and an otolaryngology attending physician. First- and second-year medical students were divided into eight groups of eight to nine medical students that rotated through different surgical skills stations and were invited to complete pre- and post-surveys before and after all eight rotations (Appendix 1). Fifty-nine students in total participated (37 were first-year and 22 were second-year medical students).

Participants opted-in to complete a pre-survey prior to the start of the first rotation and a post-survey completed after the workshop had concluded. The surveys measured the difference in self-evaluated proficiency levels across multiple surgical skills stations, including suturing and knot-tying skills, basic laparoscopy skills, endotracheal tube insertion, Foley catheter insertion, needle venipuncture, nasogastric tube insertion, IUD insertion, and sterile scrubbing technique. The pre-survey assessed the students’ initial motivation for pursuing a career in surgery. A five-point Likert scale was included to assess the students’ level of comfortability in the following surgical skills: (1) suture techniques, (2) handling laparoscopic instruments, (3) navigating a laparoscopic camera, (4) performing tasks with laparoscopic tools, (5) performing aseptic scrubbing procedures, (6) properly donning a sterile surgical gown, (7) properly donning sterile gloves, (8) inserting a nasogastric tube, (9) inserting an endotracheal tube, (10) inserting an IUD, (11) venipuncture technique, (12) Foley catheter insertion in a male patient, and (13) Foley catheter insertion in a female patient. The post-survey then asked students regarding changes in their interest in a surgical career as well as if the surgical skills workshop met their expectations in enhancing their surgical skills, which was reinforced by the use of the same Likert scale included in the pre-survey. The survey was developed by the authors; no formal validation was performed. This program evaluation was granted exemption by the UTRGV IRB.

Station set-up

Participants rotated through eight hands-on clinical skills stations led by attending physicians and resident physicians from multiple surgical specialties. Each station included a demonstration, followed by supervised deliberate practice using task trainers and simulation models. The students participated in each station for 25 minutes. Verbal instruction and individualized feedback were provided at all stations.

Station 1: Suturing Techniques

A general surgeon conducted a suturing workshop in which students learned simple interrupted sutures and surgical knot-tying. Students practiced placing up to four sutures on a synthetic suturing pad while receiving real-time feedback.

Station 2: Laparoscopic Skills

General surgery residents facilitated four laparoscopic training stations using laparoscopic box trainers with movable cameras. Students practiced camera navigation and instrument handling through simulated tasks, including transferring beans between containers and aligning plastic letters on a template.

Station 3: Nasogastric Tube Insertion

A general surgery resident demonstrated proper nasogastric tube insertion using task trainers. Students then performed the procedure on simulation models with direct supervision and feedback.

Station 4: Intubation

An otolaryngology attending physician instructed students on the indications, anatomy, and technique for endotracheal intubation. Students practiced intubation using airway mannequins under supervision.

Station 5: Intrauterine Device (IUD) Insertion

An obstetrics and gynecology attending physician demonstrated IUD insertion on pelvic models, including a review of indications and relevant anatomy. Students subsequently practiced the procedure with feedback.

Station 6: Venipuncture

A general surgeon demonstrated venipuncture and blood collection techniques using arm models filled with simulated blood. Students practiced needle insertion and tube collection and disposed of sharps in appropriate containers.

Station 7: Foley Catheter Insertion

A urology resident demonstrated sterile Foley catheter insertion using both male and female catheterization models. Students practiced the procedure on both models under supervision.

Station 8: Surgical Scrub-In Technique

General surgery residents instructed students in proper surgical scrub-in technique. Students performed surgical hand scrubbing with antiseptic solution and donned sterile gowns, gloves, and caps with assistance from residents acting as scrub nurses.

Survey design

A pre- and post-intervention survey conducted through Qualtrics (Qualtrics, Provo, Utah, United States) was administered to participants to assess self-reported familiarity and comfort with operating room (OR)-related skills and concepts. The surveys were distributed electronically and completed voluntarily and anonymously. The pre-intervention survey collected baseline demographic information, prior OR exposure (e.g., number of times present in an operating room), and self-assessed confidence in surgical skills. Both the pre- and post-intervention instruments included 13 items evaluating participants’ familiarity and comfort with specific surgical skills, instruments, and OR practices. Responses to these items were measured using a five-point Likert scale, with higher scores indicating greater familiarity or comfort.

The post-intervention survey additionally included items assessing participants’ perceptions of the educational experience and changes in confidence. All responses were recorded electronically and exported for analysis. Only completed surveys were included. Pre- and post-intervention responses were compared to evaluate changes in participants’ perceived knowledge, familiarity, and confidence following the intervention.

Statistical analysis

Descriptive data were reported as means and variances to summarize categorical responses. An unequal variance two-sample t-test was used to compare pre- and post-workshop scores regarding student comfort level with various technical skills. A unpaired t-test was conducted due to an inability to pair the pre- and post-responses. A p-value of less than 0.01 was considered statistically significant and set for all hypothesis tests. All data analysis was conducted using Microsoft Excel (version 2024; Microsoft Corporation; Redmond, Washington, United States). No missing data were present in completed surveys. The surveys were generated in Qualtrics to protect student privacy and minimize bias.

## Results

**Figure 1:**
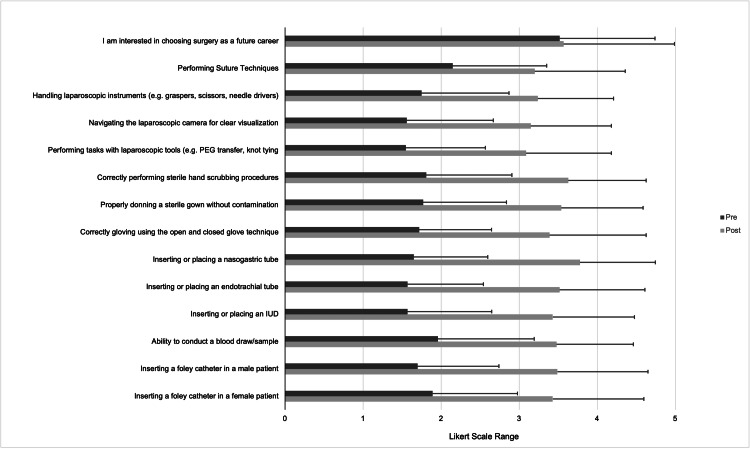
Means of Each Question on a Likert Scale, Pre- and Post-Event Pre- and post-workshop self-reported confidence and interest in surgical skills. Mean Likert scale scores (± standard error) are shown for participants’ interest in pursuing a surgical career and confidence in performing a range of procedural and perioperative skills before (dark gray) and after (light gray) participation in the surgical skills workshop. Skills assessed included suturing, laparoscopic instrument handling and camera navigation, sterile technique (hand scrubbing, gowning, and gloving), nasogastric tube placement, endotracheal intubation, IUD placement, blood draws, and Foley catheter insertion. Statistically significant improvements between pre- and post-workshop scores are indicated by an asterisk (*P < 0.05). IUD: intrauterine device.

A total of 51 medical students completed the pre-survey, and 46 medical students completed the post-survey. Students who participated in the workshop but did not opt in for survey completion were excluded from the study. On completion of the workshop, the students showed no difference in their interest in surgery (from 3.52 ± 1.22 to 3.57 ± 1.42; P = 0.88) (Table 1; Figure 1). Students showed increased confidence in performing suture techniques (from 2.15 ± 1.20 to 3.20 ± 1.17; P < 0.01), handling laparoscopic instruments (e.g., graspers, scissors, needle drivers) (from 1.75 ± 1.12 to 3.24 ± 0.97; P < 0.01), navigating the laparoscopic camera for clear visualization (from 1.56 ± 1.11 to 3.15 ± 1.03; P < 0.01), performing tasks with laparoscopic tools (e.g., percutaneous endoscopic gastrostomy (PEG) transfer, knot tying) (from 1.55 ± 1.02 to 3.09 ± 1.09; P < 0.01), correctly performing sterile hand scrubbing procedures (from 1.81 ± 1.10 to 3.63 ± 1.00; P < 0.01), properly donning a sterile gown without contamination (from 1.77 ± 1.07 to 3.54 ± 1.05; P < 0.01), correctly gloving using the open and closed glove technique (from 1.72 ± 0.93 to 3.39 ± 1.24; P < 0.01), inserting or placing a nasogastric tube (from 1.65 ± 0.95 to 3.78 ± 0.96; P < 0.01), inserting or placing an endotracheal tube (from 1.57 ± 0.97 to 3.52 ± 1.09; P < 0.01), inserting or placing an IUD (from 1.57 ± 1.08 to 3.43 ± 1.05; P < 0.01), ability to conduct a blood draw/sample (from 1.96 ±1.23 to 3.48 ±0.98; P < 0.01), inserting a Foley catheter in a male patient (from 1.70 ± 1.04 to 3.49 ± 1.16; P < 0.01), and inserting a Foley catheter in a female patient (from 1.89 ± 1.09 to 3.43 ±1.17; P < 0.01). The results of each question that was asked are shown in Figure 1.

**Table 1:** Pre- and Post-Workshop Self-Reported Confidence in Procedural Skills and Interest in Surgical Careers Among Preclinical Medical Students Legend: Values are presented as mean ± standard deviation (SD). P values represent comparisons between pre- and post-workshop responses. * indicates statistical significance (P < 0.05). IUD = intrauterine device; SD = standard deviation; PEG = percutaneous endoscopic gastrostomy.

Survey Questions	Pre	Post	P
I am interested in choosing surgery as a future career	3.52 ± 1.22	3.57 ± 1.42	0.88
Performing suture techniques	2.15 ± 1.20	3.20 ± 1.17	<0.01*
Handling laparoscopic instruments (e.g., graspers, scissors, needle drivers)	1.75 ± 1.12	3.24 ± 0.97	<0.01*
Navigating the laparoscopic camera for clear visualization	1.56 ± 1.11	3.15 ± 1.03	<0.01*
Performing tasks with laparoscopic tools (e.g., PEG transfer, knot tying)	1.55 ± 1.02	3.09 ± 1.09	<0.01*
Correctly performing sterile hand scrubbing procedures	1.81 ± 1.10	3.63 ± 1.00	<0.01*
Properly donning a sterile gown without contamination	1.77 ± 1.07	3.54 ± 1.05	<0.01*
Correctly gloving using the open and closed glove technique	1.72 ± 0.93	3.39 ± 1.24	<0.01*
Inserting or placing a nasogastric tube	1.65 ± 0.95	3.78 ± 0.96	<0.01*
Inserting or placing an endotracheal tube	1.57 ± 0.97	3.52 ± 1.09	<0.01*
Inserting or placing an IUD	1.57 ± 1.08	3.43 ± 1.05	<0.01*
Ability to conduct a blood draw/sample	1.96 ±1.23	3.48 ±0.98	<0.01*
Inserting a Foley catheter in a male patient	1.70 ± 1.04	3.49 ± 1.16	<0.01*
Inserting a Foley catheter in a female patient	1.89 ± 1.09	3.43 ±1.17	<0.01*

## Discussion

This study demonstrates that a single, organized surgical skills workshop significantly improved first- and second-year medical students’ self-reported confidence across a wide variety of technical and procedural skills. Unlike these studies, which demonstrated increased career interest, our study found no significant change in career interest (3.52 to 3.57, P = 0.88), likely due to self-selection bias and ceiling effects among our voluntarily enrolled participants [2,3,10]. Nearly all skill-based survey items showed large and statistically significant increases from pre- to post-workshop assessment (P < 0.01), indicating that brief, peer-assisted, hands-on exposure can meaningfully enhance perceived surgical technical capabilities among early medical students. Such exposure may also reduce anxiety when performing procedures during medical school clerkships [11-14].

The most pronounced improvements were observed in procedures that are typically unfamiliar to preclinical students, including nasogastric tube placement, sterile hand scrubbing and gowning, endotracheal intubation, and laparoscopic skills. These findings suggest that limited exposure, rather than inherent procedural difficulty, may represent a primary barrier to student confidence in performing procedural tasks. By providing guided instruction, immediate feedback, small-group learning environments, and low-stakes practice opportunities, the workshop effectively addressed this gap.

All skills introduced in the workshop require continuous, longitudinal practice to achieve mastery and maintain proficiency [15,16]. However, prior studies have indicated that medical students can retain laparoscopic surgical skills developed through short-term exposure [17]. Laparoscopic skills encompass multiple domains of aptitude, including instrument handling, camera navigation, and task execution. Given the increasing emphasis on minimally invasive, laparoscopic-assisted procedures in modern medicine, early exposure and familiarity with laparoscopic instruments may confer an advantage during clinical clerkships by enabling greater participation in the operating room. The significant increase in self-reported confidence in using laparoscopic tools supports simulation-based education as an effective strategy for developing psychomotor and hand-eye coordination skills prior to clinical clerkships. Although long-term skill retention was not evaluated in this study, the results are consistent with previous research demonstrating that early introduction to laparoscopic skills during medical training improves surgical technical proficiency [2,15,18,19].

In contrast to the substantial gains in technical confidence, interest in pursuing a surgical career did not significantly change following the workshop. This finding differs from prior studies reporting significant increases in medical student interest in surgical careers after participation in short-term surgical skills workshops, with interest levels rising from baseline rates of 29%-56% to post-workshop rates of 72%-88% [2,3,13,20,21]. The workshop was a voluntary, extracurricular event for which students self-registered, likely introducing substantial self-selection bias. Students with preexisting interest in surgery may have been more inclined to participate, which may explain both the relatively high baseline interest in surgical careers among attendees and the absence of significant change in post-workshop career interest. Therefore, the workshop may have served more to reinforce or consolidate existing surgical interest than to generate new interest among previously undecided students.

This limitation also affects the generalizability of the findings to the broader medical student population, particularly students with limited prior exposure to or interest in surgery. Because the participant pool may not fully represent the diversity of career interests present within an entire medical school class, the effect of similar workshops on less surgically inclined students remains unclear. To more accurately assess the impact of surgical skills workshops on future career interest, incorporating such experiences into the preclinical curriculum as a mandatory activity involving a broader, more representative student cohort may be warranted.

This study has several limitations. It was conducted at a single institution with a relatively small sample size, which may limit the generalizability of the findings to broader medical student populations. Additionally, the workshop was voluntary and extracurricular, introducing substantial self-selection bias. Students with preexisting interest in surgery may have been more likely to participate, potentially contributing to the high baseline interest in surgical careers observed among participants and limiting the ability to detect meaningful changes in career interest following the intervention.

Outcomes were based exclusively on self-reported confidence measures rather than objective assessments of procedural competency. Although participants reported significant increases in confidence, confidence does not necessarily correlate with technical proficiency, and post-workshop self-assessments may have been inflated by immediate exposure effects, social desirability bias, or enthusiasm following participation. The potential disconnect between perceived confidence and actual procedural ability should therefore be considered when interpreting these findings. Incorporation of validated assessment tools, such as Objective Structured Assessment of Technical Skills (OSATS) scoring systems or procedure-specific competency checklists, would strengthen future evaluations by providing objective measures of skill acquisition. Additionally, the survey instrument used in this study was not formally validated.

The study also lacked a control or comparison group, making it difficult to definitively attribute observed confidence gains solely to participation in the workshop rather than concurrent educational experiences. Furthermore, pre- and post-workshop surveys were administered on the same day, which may have amplified perceived confidence gains without reflecting durable learning or long-term retention. No longitudinal follow-up was performed to determine whether confidence improvements persisted over time, translated into improved clinical performance during clerkships, or increased procedural participation in surgical rotations.

Logistical variability also represented a limitation. The sterile hand scrubbing and gowning station frequently exceeded the allotted station time, resulting in inconsistent exposure duration between participant groups. Future iterations may benefit from extended station timing, improved scheduling organization, and repeated workshop sessions throughout the academic year to better assess skill retention. Future studies should additionally compare mandatory versus voluntary workshop participation models to better evaluate the impact of selection bias and determine whether these interventions influence a broader and more representative population of preclinical medical students.

## Conclusions

The intent of this study was to cultivate interest in the surgical specialties among medical students through engagement with surgical skill practice in an educational environment. This simulation-based workshop significantly improved preclinical students’ self-reported confidence across a wide range of procedural skills. While increased confidence does not equate to objective procedural competence, enhancing early-career confidence may reduce anxiety and increase willingness to participate during formal clinical clerkships. Following participation in hands-on procedural stations, students demonstrated a significant positive trend in perceived skill confidence, suggesting increased readiness for future clinical learning opportunities and engagement in surgical education.

However, the educational impact of these findings should be interpreted cautiously, as the study evaluated perceived confidence rather than objective technical ability, and no long-term follow-up was performed to assess skill retention or sustained educational outcomes. Future iterations should incorporate validated objective performance measures, such as procedural competency checklists or OSATS-based assessments, as well as longitudinal follow-up during clinical clerkships to determine whether these confidence gains translate into improved procedural competence, increased clinical participation, and sustained interest in surgical specialties.

## Appendices

Appendix 1

Pre-Survey

1) What year of medical school are you in?

Answer the following statements regarding your experience (Scale: 1-5, with 1 being the lowest and 5 being the highest rating):

2) I am interested in choosing surgery as a future career.

Answer the following statements regarding your experience (Scale: 1-5, with 1- Strongly Disagree, 2- Disagree, 3- Neutral, 4- Agree, and 5- Strongly Agree):

3) I am comfortable with the following tasks before the workshop:  

4) Performing suture techniques

5) Handling laparoscopic instruments (e.g., graspers, scissors, needle drivers)

6) Navigating the laparoscopic camera for clear visualization

7) Performing tasks with laparoscopic tools (e.g., PEG transfer, knot tying)

8) Correctly performing sterile hand scrubbing procedures

9) Properly donning a sterile gown without contamination

10) Correctly gloving using the open and closed glove technique

11) Inserting or placing a nasogastric tube

12) Inserting or placing an endotracheal tube

13) Inserting or placing an IUD

14) Ability to conduct a blood draw/sample

15) Inserting a Foley catheter in a male patient

16) Inserting a Foley catheter in a female patient

Post-Survey

1) What year of medical school are you in?

Answer the following statements regarding your experience (Scale: 1-5, with 1 being the lowest and 5 being the highest rating):

2) Overall enjoyment

3) Estimated benefit

Answer the following statements regarding your experience (Scale: 1-5, with 1- Strongly Disagree, 2- Disagree, 3- Neutral, 4- Agree, and 5- Strongly Agree):

4) I am interested in choosing surgery as a future career.

5) Participating in surgical workshops like this one will help me in my future. 

Answer the following statements regarding your experience (Scale: 1-5, with 1- Strongly Disagree, 2- Disagree, 3- Neutral, 4- Agree, and 5- Strongly Agree):

6) I am comfortable with the following tasks after the workshop: 

7) Performing suture techniques

8) Handling laparoscopic instruments (e.g., graspers, scissors, needle drivers)

9) Navigating the laparoscopic camera for clear visualization

10) Performing tasks with laparoscopic tools (e.g., PEG transfer, knot tying)

11) Correctly performing sterile hand scrubbing procedures

12) Properly donning a sterile gown without contamination

13) Correctly gloving using the open and closed glove technique

14) Inserting or placing a nasogastric tube

15) Inserting or placing an endotracheal tube

16) Inserting or placing an IUD

17) Ability to conduct a blood draw/sample

18) Inserting a Foley catheter in a male patient

19) Inserting a Foley catheter in a female patient
